# Exploring Individual- to Population-Level Impacts of Disease on Coral Reef Sponges: Using Spatial Analysis to Assess the Fate, Dynamics, and Transmission of *Aplysina* Red Band Syndrome (ARBS)

**DOI:** 10.1371/journal.pone.0079976

**Published:** 2013-11-14

**Authors:** Cole G. Easson, Marc Slattery, Henrique G. Momm, Julie B. Olson, Robert W. Thacker, Deborah J. Gochfeld

**Affiliations:** 1 Environmental Toxicology Research Program, University of Mississippi, University, Mississippi, United States of America; 2 National Center for Natural Products Research, University of Mississippi, University, Mississippi, United States of America; 3 Department of Pharmacognosy, University of Mississippi, University, Mississippi, United States of America; 4 Department of Geosciences, Middle Tennessee State University, Murfreesboro, Tennessee, United States of America; 5 Department of Biological Sciences, University of Alabama, Tuscaloosa, Alabama, United States of America; 6 Department of Biology, University of Alabama at Birmingham, Birmingham, Alabama, United States of America; University of Connecticut, United States of America

## Abstract

**Background:**

Marine diseases are of increasing concern for coral reef ecosystems, but often their causes, dynamics and impacts are unknown. The current study investigated the epidemiology of *Aplysina* Red Band Syndrome (ARBS), a disease affecting the Caribbean sponge *Aplysina cauliformis*, at both the individual and population levels. The fates of marked healthy and ARBS-infected sponges were examined over the course of a year. Population-level impacts and transmission mechanisms of ARBS were investigated by monitoring two populations of *A. cauliformis* over a three year period using digital photography and diver-collected data, and analyzing these data with GIS techniques of spatial analysis. In this study, three commonly used spatial statistics (Ripley’s K, Getis-Ord General G, and Moran’s Index) were compared to each other and with direct measurements of individual interactions using join-counts, to determine the ideal method for investigating disease dynamics and transmission mechanisms in this system. During the study period, Hurricane Irene directly impacted these populations, providing an opportunity to assess potential storm effects on *A. cauliformis* and ARBS.

**Results:**

Infection with ARBS caused increased loss of healthy sponge tissue over time and a higher likelihood of individual mortality. Hurricane Irene had a dramatic effect on *A. cauliformis* populations by greatly reducing sponge biomass on the reef, especially among diseased individuals. Spatial analysis showed that direct contact between *A. cauliformis* individuals was the likely transmission mechanism for ARBS within a population, evidenced by a significantly higher number of contact-joins between diseased sponges compared to random. Of the spatial statistics compared, the Moran’s Index best represented true connections between diseased sponges in the survey area. This study showed that spatial analysis can be a powerful tool for investigating disease dynamics and transmission in a coral reef ecosystem.

## Introduction

Substantial impacts on marine populations and communities have been attributed to diseases of marine organisms [1], [2]. Much of the marine disease literature has focused on hard corals, which have experienced massive declines in recent decades. In most cases, coral diseases are believed to be caused by microorganisms, but the specific pathogen has only been identified in a few cases [3]–[8]. In general, the understanding of marine diseases lags behind terrestrial diseases based on functional knowledge and techniques of investigation; however, this lag is particularly striking when considering the increasing rate at which marine diseases are reported [5], [9]. With coral cover declining, diseases of sponges have gained increasing attention [9]–[18]. One such disease is *Aplysina* Red Band Syndrome (ARBS) [9], an infectious disease of branching sponges in the genus *Aplysina*, characterized by an advancing red band surrounding necrotic lesions that become colonized by filamentous algae. ARBS causes physiological impairments to the host sponge, including a loss of photosymbiotic cyanobacteria, reduced growth and tissue necrosis [9], [18]. ARBS has been recorded throughout the Caribbean at prevalence rates as high as 15% of the individuals within a population [9], [18]. This disease can result in partial or total mortality of sponges that it infects, and while it is able to spread through experimental contact, the main mechanism of transmission within a natural population remains unknown [18].

In addition to disease, periodic storm events can have major impacts on coral reefs. Strong storms do not affect all reef species equally [19], and in some cases, may have positive effects on coral reefs. For example, storms promote diversity by opening new substrate for larval recruitment, and by decreasing the abundance of faster growing branching coral species, allowing slowly growing, more robust coral species to survive [20]–[22]. While storms have historically had many positive effects on coral reefs, recent studies have suggested that these storms have increased in intensity and will continue to do so under conditions of rising sea surface temperatures [23], [24]. In addition, these storms are affecting reefs that are already impacted by stressors such as disease, overfishing, and anthropogenic nutrients, which have reduced the structural and functional diversity of Caribbean reefs [19]. When these stressors are coupled with strong storms, the consequences to coral reefs can be dramatic [25].

Spatial analysis offers a powerful method to study the epidemiology of diseases within a population. Population monitoring can be used to develop a time series of disease status in individuals within a population. These spatial and temporal patterns of disease incidence can then be used to discern the process of transmission [26]. This study used spatial analysis techniques to investigate the dynamics of ARBS in two populations of *Aplysina cauliformis* on Bahamian patch reefs. ARBS presents a unique opportunity to investigate transmission mechanisms because it occurs on sponges that grow either upright or horizontally, and are able to physically contact neighboring individuals [9]. These growth strategies enabled us to evaluate three potential mechanisms of disease spread within our sponge populations: contact-driven, waterborne, and vector-driven transmission. While forced physical contact spreads this disease efficiently in both laboratory [9] and field experiments (Gochfeld, unpublished data), additional or alternative transmission mechanisms may be important on the reef. This study analyzed distribution patterns of ARBS over a three year period to determine a transmission mechanism for this disease, and compared three hypotheses of transmission [i.e., 1) contact, 2) water-borne, and 3) vector-driven transmission] using three spatial statistics methods to assess which one best represented true spatial relationships among individuals on the reef. In addition, this study investigated the impacts of a severe storm event (Hurricane Irene: Category 3; 27 August 2011) on the *A. cauliformis* populations and ARBS infections.

## Materials and Methods

### Study sites and species

This study was conducted on two shallow reefs (3–5 m) near the Perry Institute for Marine Science on Lee Stocking Island, Exuma Cays, Bahamas, from January 2008 to June 2012. Field monitoring was conducted at Big Point (N 23° 47.301”, W 76° 08.118”) and Rainbow Gardens (N 23° 47.798”, W 76° 08.786”), located 1.5 kilometers apart. Permission for use of the study locations was provided by the Department of Marine Resources, Ministry of Agricultural and Marine Resources of the Bahamas.

**Figure 1:**
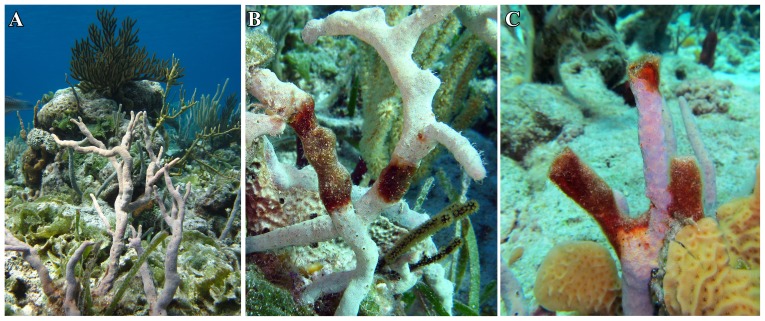
Images representing three common conditions for *A. cauliformis* individuals on the reef. **A.** Healthy *Aplysina cauliformis*. **B.** *Aplysina cauliformis* individual infected with *Aplysina* Red Band Syndrome (ARBS) **C.** Breakage of *A. cauliformis* within an ARBS lesion

The study investigated the epidemiology of ARBS in the common Caribbean branching sponge, *Aplysina cauliformis* (Figure 1A). This sponge provides essential habitat and food for many reef organisms throughout the Caribbean and is found at densities of up to 6.5 individuals m^−2^ on patch reefs in the Bahamas (Easson and Gochfeld, unpublished data). *A. cauliformis* harbors dense populations of the sponge-specific cyanobacterium *Synechococcus spongiarum*, and these populations can provide up to 75% of the sponge’s energy budget [27], [28]. In addition to *S. spongiarum*, *A. cauliformis* hosts a diverse microbial community [9], [29], [30]. Like most sponges, *A. cauliformis* produces numerous secondary metabolites [31], [32] that exhibit allelopathic, antimicrobial and feeding deterrent activity [33], [34]. Infection of *A. cauliformis* with ARBS (Figure 1B) leads to a reduction in sponge growth [18] and photosymbiont activity [18], increased chance of sponge breakage (Figure 1C), as well as changes in microbial communities [29] and sponge chemistry [34], suggesting that ARBS could lead to detrimental effects on sponge fitness and survival.

### Monitoring of individually marked sponges

In order to track the rate of progression of ARBS in individual sponges, 18 diseased individuals and their nearest healthy neighbors were marked and monitored from 2008–2009 at Big Point. Marked sponges were photographed, number of lesions counted, and measurements were made of the healthy tissue, active red bands and necrotic tissue during March 2008, July 2008 and June 2009. These data were analyzed for differential fates by indicating health status based on the presence of ARBS, as has been done in other studies investigating qualitative effects of a treatment [35]. Health status rankings were: healthy  =  1, diseased  =  2 and missing  =  3 (Wilcoxon Rank-Sum test). Healthy tissue length was compared for healthy and diseased individuals at each time (repeated measures ANOVA).

### Sponge population monitoring

Permanent 10×10 m grids were established at Big Point and Rainbow Gardens reefs. Within each grid, digital images representing 1 m^2^ were taken using a Canon D10 underwater camera, resulting in 100 images per grid. The location of each individual *Aplysina cauliformis* (≥ 5 cm total length) within each 1 m^2^ block was recorded *in situ* on an underwater paper map, total sponge length was measured using a fiberglass measuring tape, and sponge health was assessed. If the sponge was diseased, the number of lesions was counted. Grids were sampled yearly in May/July from 2010–2012, and one month after hurricane Irene (27 August 2011) in September 2011. Big Point was sampled twice in 2011 in both May and July. Each grid contained between 133 and 340 *A. cauliformis* individuals.

**Figure 2:**
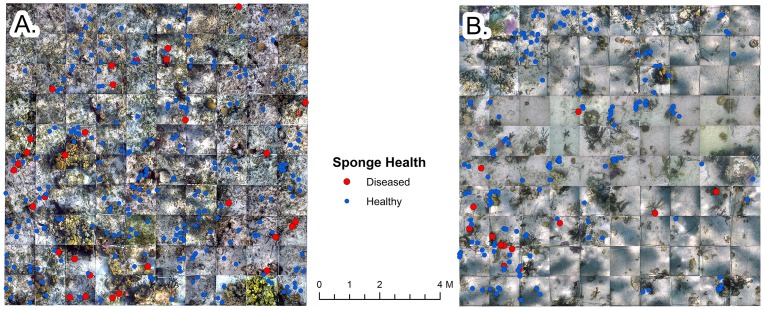
Representative photo-mosaics of the *Aplysina cauliformis* population in 10×10 m grids at Big Point (top) and Rainbow Gardens (bottom). The relative locations of healthy (blue dots) and diseased (red dots) *A. cauliformis* are shown in the grids.

Photographs from May 2011 for Big Point (BP) and July 2011 for Rainbow Gardens (RG) were georeferenced using GPS coordinates to their locations on the patch reefs and assembled into a mosaic representing the 10×10 m grid in the program ArcMap (Figure 2). Photographs from other time points were photographically georeferenced to these images at the two sites. A point vector file was then created for the sponges that were measured in each grid. Each point represented one sponge and was referenced to an attribute table containing records of its length, health, number of lesions and number of branches. The number of branches for each sponge was determined from the photographs. This process was repeated for each time point.

As a comparison to previous studies that used broader surveys to investigate marine diseases [5], [36]–[38], the 10×10 m grids containing the point vectors at each site were transformed into polygons. Each polygon measured 0.5 m^2^, and its attributes included the total number of sponges as well as the number of diseased sponges within that polygon.

To investigate whether ARBS disproportionally affected certain size classes of sponges within the population, size frequency distributions of healthy and diseased sponges were compared using a Kolmogorov-Smirnov test.

### Spatial analysis

Spatial analysis techniques are used in many fields [39]–[41], including ecology [26], [42], [43], where they have been used to study disease transmission in terrestrial systems [26], [44]. Transmission of diseases via contact, vectors or through the medium (air or water) can be discerned through distinct spatial relationships [26], [44]. In contrast to the terrestrial environment, spatial analysis techniques have seen limited applications in marine systems [36], [37], [45]–[49], and the few studies that have used them have sampled at a resolution that is too coarse to adequately investigate disease transmission mechanisms [26], [36], [37]. To date, most marine studies that have used spatial statistics have focused on those that measure clustering of attributes, such as the Ripley’s K or the Getis-Ord General G statistics [36], [37], [46], [48]–[50]. These statistics measure the concentration of attributes in an area [51], but in epidemiological studies it may be more important to investigate relationships of individuals in the population. Less frequently used statistics, such as the Moran’s Index, capture these individual relationships by measuring spatial autocorrelation (simultaneous similarity of feature values and location) between individuals [50], [52], [53].

To assess the pattern of clustering of disease within a population of *A. cauliformis*, these three spatial statistics (Ripley’s K function, Getis-Ord General G, and Moran’s Index) were compared using the spatial analysis toolkit in the ArcGIS toolbox. Spatial patterns within the sponge populations were analyzed on each grid at each point in time. These spatial statistics were also compared to a higher resolution technique (join-counts) that investigated the connectedness of individuals within the population using two metrics: (1) Physical contact connectedness (as an indicator of direct transmission of disease) and (2) Gabriel connectedness (as an indicator of vector mediated transmission of disease [26], [44]).

Ripley’s K function is used to analyze spatial patterns and investigate spatial dependence of features (clustering or dispersion). While many spatial statistics require selection of a specific scale, the Ripley’s K function examines patterns over a range of scales to determine the appropriate one for the specific dataset [51], [54]–[56]. The Getis-Ord General G statistic measures how concentrated certain values are in a selected area. This statistic can be used to monitor high proportions of a particular attribute in an area, for example, the number of diseased sponges [51], [57]. The Moran’s I statistic measures spatial autocorrelation, which is the similarity of features based on both their locations and values. This tool measures the spatial relationship between features of similar and different values to determine patterns of clustering or dispersion in a population [51], [52], [58], [59]. Whereas the Getis-Ord statistic measures the concentration of values in an area, Moran’s I determines the spatial relationship between individuals of the same and different values.

The Ripley’s K statistic has been used in many marine disease studies [8], [36], [46], [48], [49]. Here, a weighted Ripley’s K statistic [51], [60], [61] was used to assess non-random distribution patterns within the sponge populations and to discern the scale at which these patterns occurred. The weighted Ripley’s K statistic randomly distributes an attribute (in this case, sponge health [healthy or diseased]) among the existing points in the grid. Each trial was run for 100 iterations with 100 distance bands over a scale of 10 m, resulting in each distance band measuring 10 cm. The data table containing the 10 cm distance band measurements was then examined for any areas where the observed distribution differed significantly (areas where the expected random distribution values fell outside the confidence envelope generated from the 100 iterations in the analysis) from the expected random distribution.

For the Getis-Ord General G and Moran’s I statistics, the sponges on the grid, represented by point vectors, were converted to Thiessen polygons to best represent the spatial relationships among sponges in the population [62]. Each Thiessen polygon represents the point with all its attributes and its area of influence, which is equal to one half of the distance between the point and each of its neighbors. The number of sides of the polygon reflects the number of neighbors with converging areas of influence. The Getis-Ord General G statistic was run for the attribute sponge health using calculated network spatial weights to conceptualize the spatial relationships of the sponges in the grid. No barriers within the grid were considered, so a Euclidian distance method was used. The Getis-Ord Gi*Hot Spot Analysis was used to visualize specific areas of clustering in the grid. These grid maps showed specific Thiessen polygons with significant z-scores as calculated by this statistic [51], [63]. The Moran’s Index statistic was calculated to examine global spatial autocorrelation between sponges weighted by the attribute sponge health using the same parameters employed for the Getis-Ord statistic. These relationships were mapped onto the grid to identify local scale clusters and outliers in the spatial autocorrelation analysis using the Cluster and Outlier Analysis (Anselin Local Moran’s I) tool in ArcGIS [51], [64].

To address disease transmission mechanisms between sampling times, join-counts were performed on all sponges within each population [26], [65]. Because each sponge was assigned a specific location within the grid, each sponge had a specific Euclidian distance and spatial relationship to every other sponge in the grid. From these relationships, we were able to establish the number of “joins” (connections) between individuals. Join-counts enabled us to track the suspected origin of an ARBS-affected sponge in one sampling time to a diseased sponge in an earlier sampling time [26], [44].

Join-counts in this study were used to assess the presence of two types of connectedness: physical contact and Gabriel (vector) contact. Transmission via direct physical contact between sponges was hypothesized to be important based on previous studies demonstrating ARBS transmission with forced contact between diseased and healthy individuals [9], and because the morphology of *A. cauliformis* enables potential physical contact with neighboring sponges. Gabriel connectedness represents transmission via a vector, and was adapted from a terrestrial model used to investigate transmission of a plant disease by pollinating insects [26], [44]. In this study, Gabriel connectedness was used to test the hypothesis that transmission could occur through spongivore feeding [66]. To assess physical contact connectedness in the grid, the distance between each sponge and every other sponge in the grid was calculated. Using the join tool in ArcGIS, the attributes of each individual sponge and the attributes of all of its neighbors were joined in the data table of the distances between individuals. Using the sponge length measured *in situ*, and the number of branches determined from the photographs, an algorithm was developed to account for all sponge-sponge interactions within a single sampling time. The algorithm determined sponge interactions by selecting individuals whose Euclidian distance was less than the sum of each sponge’s length divided by its number of branches. This method assumed equal branch length for each sponge, which likely underestimates sponge interactions. These interactions were grouped into four classes: total (T), healthy:healthy (H:H), healthy:diseased (H:D), and diseased:diseased (D:D) joins. This method was automated for each grid using a Python script in ArcMap to test for contact-connectedness between sponges. To test whether the frequency of these observed contacts was statistically different from a random distribution of contact frequency, another Python script was developed which took the feature layer containing all the sponges and their attributes used in calculating the observed contacts, and randomly distributed these points in a 10×10 m grid before recalculating the frequency of contact-joins within each class. One hundred iterations of this process were run [26], and the averages for each class were calculated. From these data, we were able to determine how many of the 100 random iterations overestimated, underestimated or accurately estimated the observed contacts in each of the four classes. This information was then translated into a proportion by dividing the number of realizations that contained more or an equal number of joins compared to the observed values by the total number of realizations, and this proportion became the p-value, as outlined in previous studies [26], [44], [67].

To test for vector-based transmission, we used a Gabriel connectedness scheme originally employed in studies of plant pollinators [26]. Using this scheme, two points in the grid are connected if there are no other points that occur within a circle that has a diameter equal to the distance between the points [26]. This model assumes that the vector will most likely travel to the nearest sponge, regardless of the Euclidian distance between them. In addition, it assumes that the vector only travels between sponges of a single species, because this disease only affects *A. cauliformis* at the sites studied. To determine if the observed Gabriel connections differed from random, the points were randomized, and the connections were determined in 100 iterations and compared to the observed connections, as described previously.

Join counts also allowed for calculation of a transmission rate for each potential transmission mechanism. Transmission rate was calculated as D:D / (D:D + D:H), where D:D represents the number of joins between diseased individuals and D:H represents the number of joins between a diseased individual and a healthy individual (total diseased sponge connections).

The polygon-based datasets were analyzed to identify pairs of polygons sharing boundaries and containing at least one diseased individual. The number of connections between polygons containing diseased sponges was counted for each time point. Similar to previous analyses, the original attributes describing each polygon were randomly assigned, creating a different dataset in a total of 100 iterations, and the expected random number of diseased polygon connections was compared to the observed diseased polygon connections as described previously.

## Results

### Monitoring of individually marked sponges

**Figure 3:**
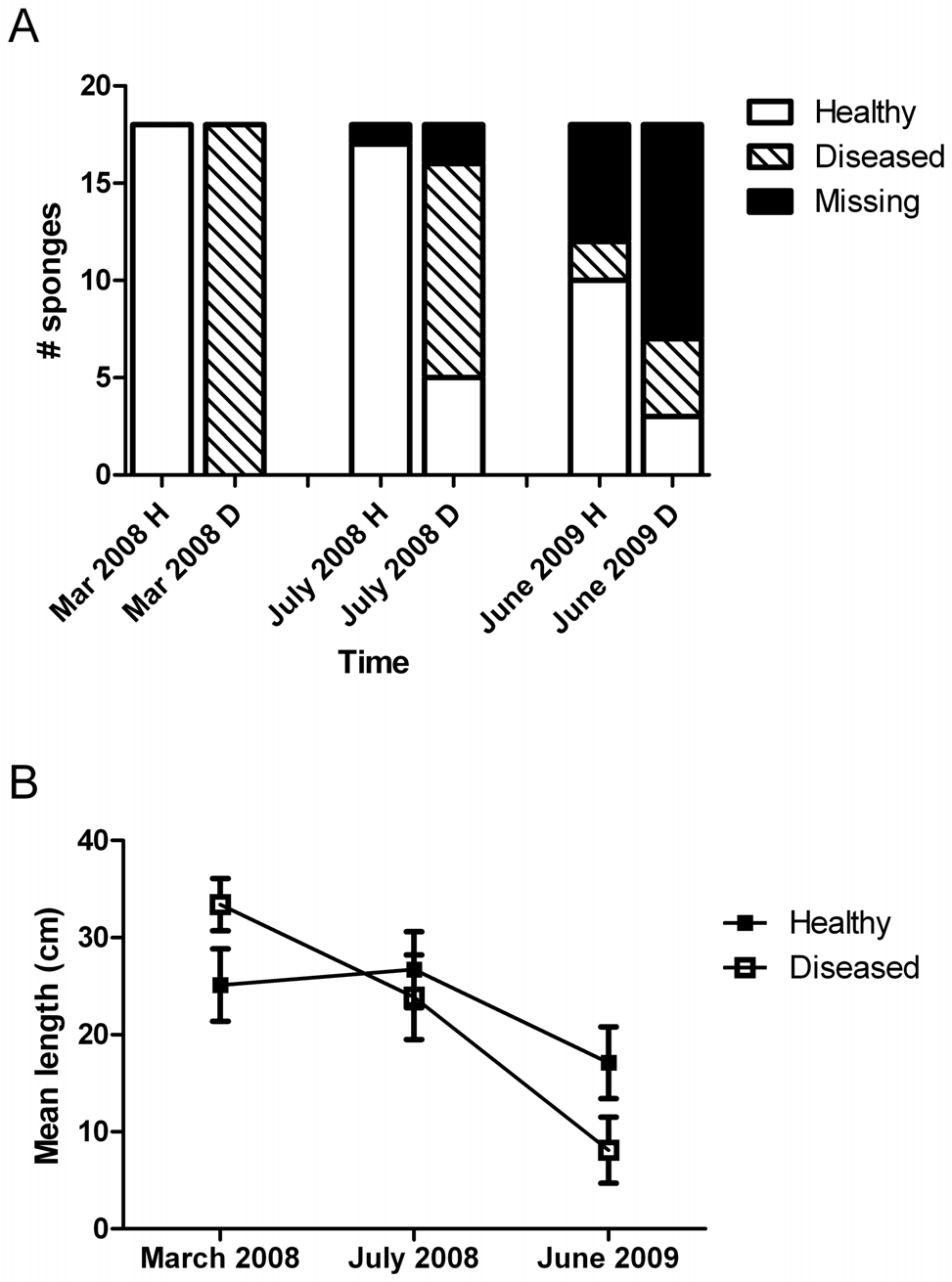
Fate and tissue loss comparisons between marked healthy and diseased *A. cauliformis* individuals. **A.** Fate of healthy and diseased marked sponges. Diseased sponges were more likely to stay diseased or go missing compared to healthy sponges (Wilcoxon rank-sum analysis, P  =  0.03. **B.** Mean (± SE) length of marked sponges over time. While the diseased sponges were larger than healthy sponges in March 2008, their mean lengths declined at a greater rate than healthy sponges (repeated measures ANOVA, P  =  0.04).

Analysis of individually marked sponges demonstrated differential fates for healthy and diseased individuals. At each subsequent sampling time, the health status of the monitored diseased individuals was significantly different than that of their healthy neighbors, mostly due to a greater number of diseased individuals going missing in surveys through time (Wilcoxon Rank-Sum test, P < 0.0001 and 0.03 for July 2008 and June 2009, respectively, Figure 3A). Initial health status did not significantly affect the length of healthy tissue through time (Figure 3B; P  =  0.11), but there was a significant change in length of healthy tissue over the course of the monitoring (P < 0.001). Length of healthy tissue was significantly influenced by an interaction between health status and time (P  =  0.04), showing that marked diseased sponges lost more biomass over the course of the monitoring compared to marked healthy sponges. Analysis of sizes at each time point showed that while diseased sponges were initially larger than healthy sponges (ANOVA: P  =  0.02), by the final sampling time there was a trend towards diseased sponges being smaller than healthy sponges (P  =  0.06).

### Sponge population monitoring

Sponge length, total length of all sponges and the number of healthy and diseased sponges from each site and sampling time point are summarized in Table 1. The proportion of diseased individuals in the population varied from year to year (3.3% – 11%); while the average sponge length remained relatively similar both between sites and time points. Big Point experienced a pronounced decline in sponge biomass, as exhibited by the reduced total sponge number and length over time. The main loss in sponge biomass on the reefs seems to be related to periodic storm events, as Hurricane Irene caused a pronounced reduction in sponge biomass on both reefs in September 2011.

**Table 1:** *Aplysina cauliformis* population attributes at two shallow reef sites in the Bahamas from 2010 to 2012.

	Total sponges	Healthy sponges	Diseased sponges	Percent Diseased	Mean±SE length (cm)	Total length (cm)
Big Point May 2010	342	330	12	3.5	52±4	17,795
Big Point May 2011	285	254	31	10.9	46±4	13,063
Big Point July 2011	270	243	27	10	47±4	12,728
Big Point September 2011	280	261	19	6.8	38±3	10,633
Big Point June 2012	320	305	15	4.7	36±2	11,529
Rainbow Gardens May 2010	133	126	7	4	56±6	7,400
Rainbow Gardens July 2011	187	174	13	9.8	48±4	8,283
Rainbow Gardens September 2011	118	108	10	8.5	54±5	6,405
Rainbow Gardens June 2012	152	147	5	3.3	50±5	7,599

The dashed line represents the occurrence of Hurricane Irene.

**Figure 4:**
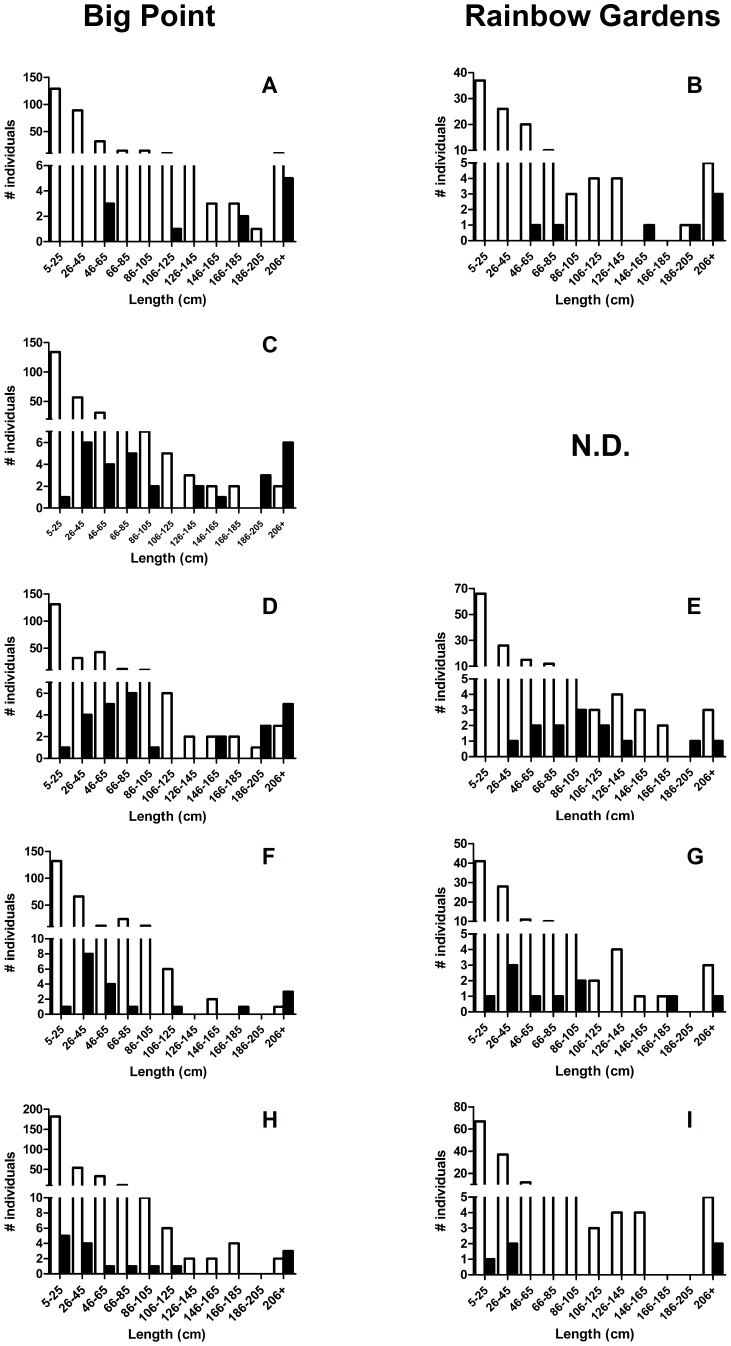
Size frequency distribution of healthy and diseased sponges in the grids at Big Point and Rainbow Gardens. A-E are pre-storm sampling times and F-I are post-storm sampling times. White bars represent healthy sponges and black bars represent diseased sponges.

ARBS disproportionately affected larger sponges in the population, as determined by comparing the size frequency distributions of healthy and diseased sponges (Figure 4). All pre-hurricane grids showed a significant difference in the size frequency distribution of healthy and diseased sponges (Kolmogorov-Smirnov: P  =  0.003). The size-frequency distributions of healthy and diseased sponges remained significantly different after the hurricane at Big Point (P  =  0.01), but became more similar at Rainbow Gardens (P  =  0.4). This shift in size at Rainbow Gardens is indicative of increased breakage of diseased sponges compared to healthy sponges. While many large sponges in both grids were healthy, diseased sponges were usually in the larger size range of sponges in the population. Although it is possible that ARBS targets large sponges, it is more likely that these sponges are affected at a higher frequency because they are older, which increases their probability of coming in contact with the pathogen. Alternatively, larger sponges have longer branch lengths and are able to interact with more sponges, thereby increasing their chances of interacting with other diseased individuals within the survey area.

### Spatial analysis

**Ripley’s K Function.** This analysis showed clustering of ARBS at all sites and time points. Most grids showed only slight clustering compared to an expected random distribution. Big Point, where the *A. cauliformis* population was denser, showed clustering of diseased sponges at a scale between 0.3 and 0.5 m, with the exception of the May 2010 time point, which showed a much larger maximum clustering scale of 5.5 m. Rainbow Gardens showed maximum clustering at 2–3 m scale for all time points.

**Figure 5:**
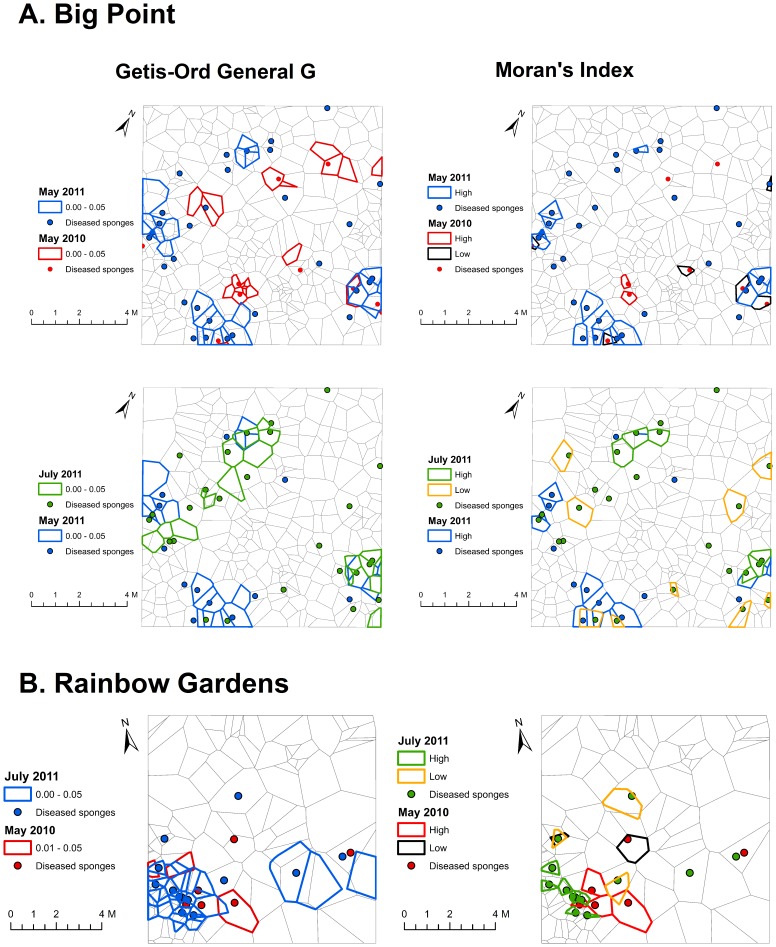
Comparison of areas of clustering and dispersion between the Getis-Ord General G and Moran’s I statistics. Getis-Ord quadrats (on left) display areas where the General G value was significant for clustering. Moran’s I quadrats (on right) display areas of significantly high clustering and significantly low clustering. These statistics recognize slightly different areas as “clustered” due to differences in the ways in which they calculate spatial relationships. Also note that between sampling times, significant clusters often overlap or are immediately adjacent to one another, suggesting that transmission occurs over a relatively small scale.

**Figure 6:**
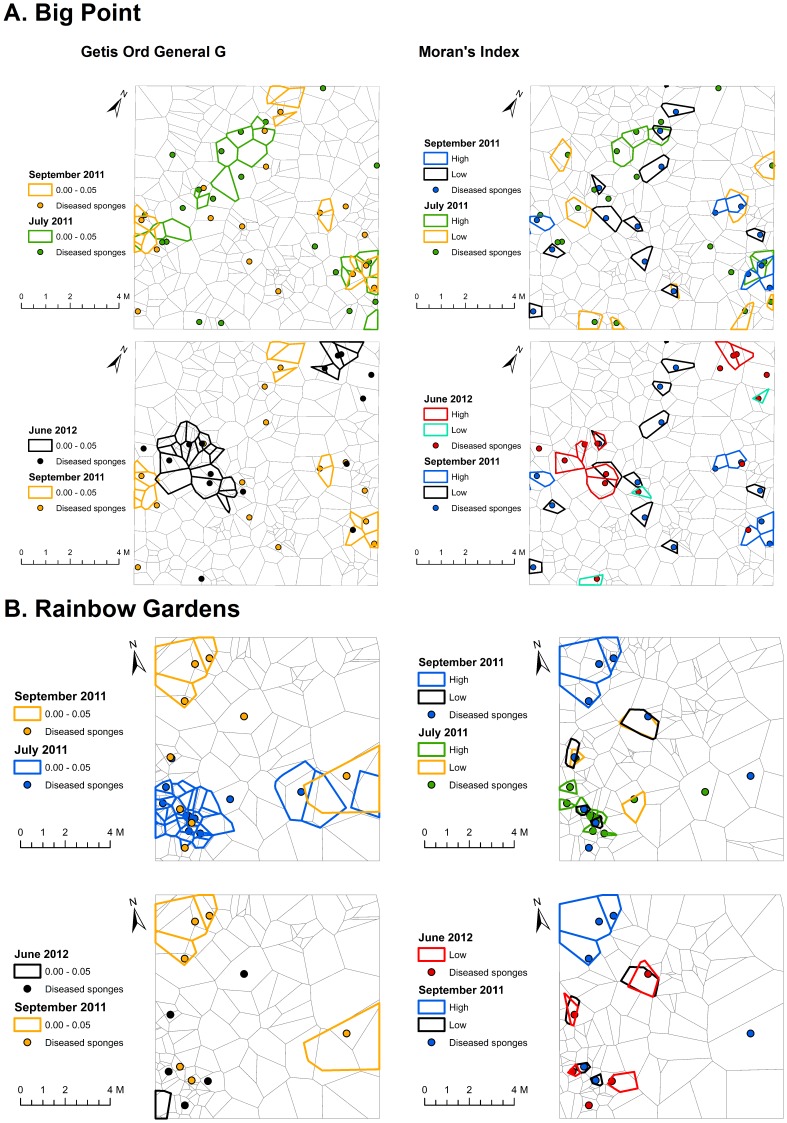
Clustering patterns of ARBS in the *Aplysina cauliformis* populations during post-storm sampling times. Getis-Ord results are shown on the left and Moran’s I results are shown on the right. The hurricane randomized the patterns observed during pre-storm sampling times, but this pattern appeared to recover to some degree between September 2011 and June 2012 at Big Point.

**Getis-Ord General G.** Significant global clustering of ARBS for the pre-storm time points of Big Point May 2011, Big Point July 2011 and Rainbow Gardens July 2011 time points (P  =  0.04, 0.02 and 0.006, respectively, Figure 5) was found, while the sponges from Big Point May 2010 and Rainbow Gardens May 2010 did not exhibit global clustering across their respective grids (P  =  0.88, 0.98, respectively). No post-hurricane time points at either site exhibited global clustering patterns (P > 0.05, Figure 6). Even though some of these grids did not exhibit global clustering patterns of ARBS, every time point at each site showed ARBS hot-spots, suggestive of some small-scale clustering within the grid (Figures 5 and 6).

**Moran’s Index.** Tests of the Moran’s I statistic revealed global spatial autocorrelation in pre-storm time points for Big Point May 2011, Rainbow Gardens May 2010, and Rainbow Gardens July 2011 (Moran’s I; P  =  0.002, 0.07, 0.0005, respectively, Figure 5). While immediate post-hurricane grids (September 2011) showed no significant spatial autocorrelation between diseased individuals, there was significant spatial autocorrelation by June 2012 at Big Point (P  =  0.02; Figure 6), but not at Rainbow Gardens (June 2012), which suggests a stronger influence of a transmission mechanism in a more dense *A. cauliformis* population. Cluster and Outlier (Anselin Local Moran’s I) analysis revealed specific areas of each grid that exhibited significantly high spatial autocorrelation, suggesting clustering, or significantly low spatial autocorrelation, suggesting dispersion of diseased sponges (Figures 5 and 6). The Cluster and Outlier analysis indicated that in time points without significant global spatial autocorrelation, the areas of significant negative spatial autocorrelation outnumbered those with significant positive spatial autocorrelation.

**Join-counts.** Join-count statistics suggested contact as the likely mechanism of transmission, demonstrated by significantly higher D:D contact joins in 3 of the 5 pre-hurricane grids (Table 2). Gabriel (vector) D:D joins did not differ from random at any sampling time, and in 60% of cases, sponges that were connected by Gabriel connectedness were also connected by contact connectedness. One exception was Rainbow Gardens in May 2010, where there were no shared joins, yet there was a trend (P  =  0.08) toward significant Gabriel connectedness. Post-hurricane grids showed no significance for either type of connectedness. While the post-hurricane grids at Big Point showed increased probability of contact connectedness from September 2011 to June 2012 (29% to 86%, respectively), the Rainbow Gardens grids showed no such trend. These data suggest the existence of a density-dependent effect, due to a higher overall density of sponges at Big Point than at Rainbow Gardens, further implicating contact as the primary mode of transmission in these populations.

**Table 2:** Join-count statistics of healthy and diseased *A. cauliformis* in 10×10 m grids at two sites in the Bahamas.

	Contact connectedness							Gabriel Connectedness					
	Observed/ Expected joins				Observed/Expected Joins				
	H:H	H:D	D:D	Total	*P = *	T. rate	H:H		H:D	D:D	Total	*P = *	T. rate
BP May 2010	873/843	98/143	2/4.4	973/991	0.93	2%	587/604		41/45	0/1	628/650	0.99	0%
BP May 2011	393/328	106/144	24/14	524/486	**0.03**	18%	407/425		100/109	10/7	517/540	0.5	9%
BP July 2011	458/331	126/156	18/14	602/502	0.3	12.5%	393/410		81/92	8/5	482/507	0.13	9%
BP September 2011	412/341	94/92	4/4.7	510/434	0.71	4%	424/459		60/67	2/2	486/529	0.7	3%
BP June 2012	482/381	72/80	6/3	560/464	0.14	8%	508/549		46/55	2/1	556/605	0.27	4%
RG May 2010	184/83	30/20	4/1	218/104	**0.05**	12%	197/216		23/24	2/0.5	222/241	0.08	8%
RG July 2011	298/121	66/29	8/1.6	373/152	**0.03**	11%	225/274		42/45	2/1.6	269/321	0.45	4.5%
RG September 2011	236/71	46/18	0/1	282/90	0.99	0%	157/176		30/33	2/1.4	189/210	0.39	6%
RG June 2012	306/119	20/12	0/0.2	326/132	0.99	0%	221/259		15/18	0/0.3	236/278	0.99	0%

Joins are represented as observed vs. expected (based on a random distribution) number of H:H  =  healthy:healthy, H:D  =  healthy:diseased, D:D  =  diseased:diseased; Total  =  total joins in a grid. P = the proportion of random diseased:diseased joins that were greater than or equal to the observed number of diseased:diseased joins. T. rate  =  transmission rate, calculated as observed D:D divided by observed D:D plus H:D. BP  =  Big Point and RG  =  Rainbow Gardens. Bold numbers  =  significant values and Underlined numbers  =  trends in the results. The dashed line represents the occurrence of Hurricane Irene.

Analysis of polygon grids demonstrated a strong dependence of these results on clustering scale. At Big Point, where maximum clustering for significant contact joins was small, the 0.5 m^2^ grids did not adequately reflect the trends seen in the join-count data for individuals. However, at Rainbow Gardens, where maximum individual clustering was at a larger scale, the same trends were observed for both significant point joins and polygon joins.

## Discussion

### Fate and Dynamics of ARBS

Previous studies have documented detrimental effects of ARBS on *A. cauliformis* [9], [18], but did not determine whether ARBS impacted the long-term survival of individuals on the reef. By monitoring individually marked sponges through time, our data demonstrate that ARBS infection increased a sponge’s rate of tissue loss over time, due to the expansion of the ARBS lesion and an increased probability of breakage. ARBS infection also increased the probability of death and removal of an individual from the reef, suggesting that infection with ARBS leads to a differential proximate fate of individual sponges on a reef.

Biomass of *A. cauliformis* populations declined over time. At Big Point, both total biomass and mean length of sponges in the population declined. At Rainbow Gardens, after an initial increase in biomass in July 2011, overall biomass decreased while mean length of sponges in the population remained relatively stable. The greatest influence on the sponge population, as well as to the coral reef community as a whole, was a major storm event. The immediate post-storm sampling time (September 2011) showed dramatic decreases in sponge biomass over a very short period of time (loss of 20.95 and 18.78 m for Big Point and Rainbow Gardens, respectively). By the following June, both populations had gained biomass and the number of individuals in the population had increased. This increase in individuals, combined with the changes in the post-storm size frequency distribution towards smaller sponges, may represent a combination of new recruits and fragmentation of *A. cauliformis* due to breakage from storm and/or disease. Fragmentation is an important mode of reproduction for some branching sponge species [68]–[70], but this increased fragmentation of individuals with active ARBS lesions during storm events could contribute to the spread of ARBS to other members of the population and even to other reefs in the region.

### ARBS transmission in a natural population

Epidemiology in marine systems has been extensively studied [2], [3], [6], [16], [17], and while some of these studies have used spatial analysis techniques [5], [36]–[38], [46], [48]–[50], [53], [71], few have used them to their full potential, resulting in a lack of mechanistic information from the data [72]. Additionally, since spatial and temporal scales of transmission patterns can vary greatly among diseases [26], [44], [46], it is imperative to have a basic understanding of disease dynamics prior to designing a relevant study to investigate disease transmission. The current study built on previous studies of ARBS and applied a variety of spatial statistical techniques to investigate clustering and specific connectedness between sponges on the reef in order to determine the most likely transmission mechanism.

The significant results of these analyses showed ARBS transmission via direct contact in three out of five pre-storm time points. Following hurricane disturbance, significant transmission by direct contact was not observed; however, these observations added support to the theory of transmission by physical contact. Previous data [9] and post-storm size frequency data showed that diseased sponges were more susceptible to breakage. When these sponges fragmented or were removed from the substrate, they could have rolled around the reef, potentially randomly contacting other sponges in the population. Using the physical contact theory, if contact with diseased individuals was randomized, the disease pattern should also be randomized, as we observed in the post-hurricane time points. In contrast, we would not expect a vector transmission to become randomized after the hurricane, because the feeding patterns of spongivores, for example *Canthigaster rostrata* [34] or *Monacanthus tuckeri* (Easson pers. obs.), would not be randomized suddenly due to the hurricane. Additionally, between September 2011 and June 2012, the probability of contact connectedness at Big Point increased from 29% to 86% as the time since the storm increased. This trend was not observed at Rainbow Gardens, where the sponge population is less dense than at Big Point. A direct contact-driven transmission mechanism would be more dependent on density of the population compared to a vector driven mechanism. Further support for contact-driven transmission is reflected in the transmission rate data: ARBS rebounded at Big Point 10 months after the hurricane, but at Rainbow Gardens, transmission rate remained low, coinciding with an absence of direct contacts between diseased sponges (Table 2).

### Spatial statistics comparison

While join-count statistics are useful for determining mechanistic information, these statistics are not commonly used in marine epidemiology studies. Direct comparison of three commonly used statistics in the current study indicates that the Moran’s Index was the most accurate at predicting significant transmission within the population of *A. cauliformis*. The Ripley’s K statistic in the current study showed some degree of clustering at all sampling times, but over a highly variable range of spatial scales (Table 3). Due to the wide clustering range at some time points, and the variability between time points, this statistic simply suggested that there might be some degree of clustering in the population. The Getis-Ord General G spatial statistic showed significant ARBS clustering at some time points, as well as specific areas of clustering within the grid for all time points. However, significant clustering using this statistic did not always correspond with significant results of actual connections (join-counts) between individuals, which suggests that measuring clustering with the Getis-Ord General G method may not represent true spatial relationships between individuals on the reef. Analysis of spatial autocorrelation using the Moran’s Index provided results that aligned best with the true individual connectedness results. In every case where the join-count connections were significant, the Moran’s I results were also significant, and this statistic seemed more sensitive to spatial relationships than the join-count connections. This is likely due to the Moran’s I statistic not directly measuring connections between diseased individuals, but focusing instead on the distance between diseased individuals, which allows the statistic to infer spatial relationships in the absence of actual contact between individuals, possibly due to the death of an intermediate sponge that may have transmitted ARBS to both currently infected individuals.

**Table 3:** Summary of spatial statistical analyses.

Statistic	Origin	What is Tested			References in Marine Epidemiology					
Ripley's K function	Ripley 1981	Clustering or dispersion over a range of distances			Jolles et al. 2002, Gardner et al. 2008, Zvuloni et al. 2009,					
					Lentz et al. 2011, Muller and van Woesik 2012					
Getis-Ord General G	Getis and Ord 1992	Clustering of values in a given area			LeDrew et al. 2004, Roff et al. 2011, Ban et al. 2012					
			
Moran's Index	Moran 1950	Clustering via measuring spatial autocorrelation:			Van Houton et al. 2010, Ban et al. 2012					
		Feature similarity based on locations and values								
Join-Count	Sokal and Oden 1978	Connections between individuals in a			Jolles et al. 2002, Zvuloni et al. 2009					
		population (Contact, Gabriel, Nearest Neighbor)								
**Results Summary**		**BP May 2010**	**BP May 2011**	**BP July 2011**	**BP Sept 2011**	**BP June 2012**	**RG May 2010**	**RG July 2011**	**RG Sept 2011**	**RG June 2012**
Ripley's K function	Max	5.5 m	0.3 m	0.3 m	0.5 m	0.3 m	3.1 m	2.3 m	2.5 m	2 m
	Range*	0–7 m	0–1.8, 2.3–6.4 m	0–1 m	0–6.2 m	0–1.7, 4.4–6.1 m	0–6 m	0–5.9 m	0–5.4 m	0–6.2 m
Getis-Ord General G	Clusters	23	31	19	17	26	8	40	4	1
	P-value	0.88	0.04	0.02	0.64	0.39	0.98	0.0006	0.59	0.53
Moran's Index	Clusters	2	15	6	7	8	4	8	3	0
	Outliers	6	10	9	12	7	2	3	4	4
	P-value	0.9	0.002	0.47	0.72	0.02	0.07	0.0005	0.95	0.147
Join-Count -	Diseased joins (O/E)	2/4	24/14	18/14	4/5	6/3	4/1	8/2	0/1	0/0
Direct Contact	P-value	0.93	0.03	0.3	0.71	0.14	0.05	0.03	0.99	0.99
Join-Count -	Diseased joins (O/E)	0/1	10/7	8/5	2/2	2/1	2/0.5	2/2	2/1	0/0
Gabriel (vector)	P-value	0.99	0.5	0.13	0.7	0.27	0.08	0.45	0.39	0.99

Comparison of the results of spatial statistics associated with disease of *Aplysina cauliformis* two populations at different time points. Each statistic tests for slightly different spatial characteristics. Contact connectedness was best able to explain clustering patterns within the study grids. The results of the Moran’s Index best represented individual connections shown by direct contact join-counts for ARBS infected sponges. *Range represents scale of significant clustering for the Ripley’s K statistic. Clusters and Outliers are individual Thiessen polygons in the grids that exhibited significant values for the Getis-Ord General G and the Moran’s Index Statistics. BP = Big Point, RG = Rainbow Gardens.

### Spatial patterns in a dynamic sponge population

Multiple factors could affect the ability to characterize transmission mechanisms within natural populations. In the case of this study, one challenge was the ephemeral nature of the sponges themselves. As seen in the marked sponge data, the annual resample rate was 67% and 39%, for healthy and diseased individuals, respectively. Unlike corals, which are long-lived (years to decades), and leave behind a permanent skeletal record of their location, sponges have shorter life-spans (months to years), and when they die, they do not leave behind a skeleton that can be accounted for in subsequent sampling times. Thus, the ability to resample individuals and determine their proximate fate is lower than in studies investigating coral diseases. Another factor that might affect a spatial population study like the current one is the influence of sponges outside the sampling area. Our grids represent only a portion of the shallow reefs on which they are located. Thus, sponges just outside the sampling area could influence disease dynamics. Many diseased sponges at these sites occurred along the edges of the grids, but because these outside sponges were never directly sampled, we can only infer their influence on sponges inside the grid. These issues are potentially reflected in some of the insignificant statistical results found in this study, suggesting that additional factors may be influencing the spatial dynamics of the sponge population in these grids.

Analysis of the 0.5 meter polygon grids showed a strong influence of clustering scale, which is likely a reflection of a lower *A. cauliformis* population density, and therefore a larger scale of clustering, at Rainbow Gardens than at Big Point. Since it appears that transmission processes did not differ between sites, the difference in clustering scale between sites is likely an artifact of this statistical method, in which sponges are analyzed as groups instead of as individuals, rather than a site difference in disease dynamics. With the resolution loss by analyzing groups of individuals and the strong influence of clustering scale, it is clear that when investigating disease transmission, analysis of connections must be investigated at the individual level of resolution.

While the individual connectedness models fit reasonably well, not all assumptions of the models were met. In the physical contact model, the sponge branch lengths were assumed to be equal because exact branch length of every sponge was not recorded, and this may underestimate sponge contacts. The vector model (Gabriel connectedness) assumed that the vectors, in this case spongivores, fed on *A. cauliformis* exclusively. While many organisms eat sponges on Caribbean reefs [73], none have been documented to feed exclusively on *A. cauliformis*. This could account for the presence of ARBS-like lesions in other species of Verongids, but it may also dilute the influence of vector-driven transmission in our model, because a spongivore may move to another sponge species after feeding on a diseased *A. cauliformis* individual. Conversely, any effects of vector transmission from other sponge species will be unaccounted for and therefore underestimated. Despite these imperfections, we feel that these statistical models adequately represent the dynamics of ARBS transmission due to a significant contact connectedness signal that shows up over the noise of a natural system.

In conclusion, ARBS is a detrimental disease to individuals, but its effects at the population level were not as obvious, possibly due to the dramatic and potentially confounding effects of a hurricane impacting our study sites. Even with this limitation, spatial analysis techniques tremendously increased our understanding of the dynamics of *Aplysina* Red Band Syndrome within *A. cauliformis* populations, and enabled us to propose a physical contact mechanism of ARBS transmission within populations of *A. cauliformis*. However, this study also raised questions regarding disease susceptibility of these sponges. Even among sponges that were in contact, transmission rates for ARBS were typically low, suggesting that some genotypes may be more susceptible to infection than others. With the use of next-generation sequencing techniques, we can begin to address whether genetic variability among individuals is correlated with susceptibility to infection. Coupling genomic techniques with spatial analysis may enable us to better understand the impacts of ARBS on *Aplysina* populations in the Caribbean.
